# An update on the epidemiology of pediatric COVID-19 in Brazil

**DOI:** 10.1590/1984-0462/2022/40/2021367

**Published:** 2021-04-04

**Authors:** Braian Lucas Aguiar Sousa, Clovis Artur Silva, Alexandre Archanjo Ferraro

**Affiliations:** a Universidade de São Paulo, São Paulo, SP, Brazil.

The coronavirus disease 2019 (COVID-19) pandemic impacted the whole world, challenging health systems, crashing economies, and taking the lives of over 4.5 million people. In a short period of time, to contain the spread of the severe acute respiratory syndrome coronavirus 2 (SARS-CoV-2) infection, the world has adapted to a new reality of quarantines or lockdowns, mask-wearing, and social distancing. No other condition in human history has been so widely studied, leading to the development of safe and effective COVID-19 vaccines in record time.

Since the beginning of the pandemic, it is clear that newborns, children, and adolescents are less impacted by this emerging condition than adults. Most of them present mild symptoms; hospitalization and death of pediatric patients are rare.^1^ This fact is highly unusual, since younger children are more vulnerable to most respiratory viruses, such as influenza or syncytial respiratory virus (SRV), with a higher risk of unfavorable outcomes. There are multiple pathophysiological mechanisms involved in SARS-CoV-2 pediatric infection, some of which could explain this contradiction. Children have fewer angiotensin-converting enzyme (ACE) receptors, leading to decreased cell infection,^2^ and pre-existing neutralizing antibodies and T-cell immunity to seasonal coronavirus might cross-protect against COVID-19.^3^ More recently, the focus has been over the role of the innate immune response in pediatric populations, and on how a less specific and more swift immune response might be implicated in protecting children.^4,5^

Despite that, children and adolescents may have poor outcomes, including severe and unique manifestations.^6^ Indeed, the recognition of the multisystem inflammatory syndrome in children (MIS-C), a life-threatening condition with high rates of morbimortality, reinforces the importance of children and adolescent protection, and the need of advancing COVID-19 research for pediatric patient populations.^7,8^

The burden of pediatric COVID-19 populations is wide, with physical, social, emotional, and learning impacts, including signs and symptoms that may be persistent and incapacitating. In this regard, long-term post-acute sequelae of SARS-CoV-2 or long COVID-19 manifestations are defined when clinical abnormalities continue after 12 weeks of the onset of acute COVID-19 and cannot be justified by other conditions. A recent report from our university hospital showed that approximately 40% of laboratory-confirmed pediatric COVID-19 survivors reported at least one persistent symptom at the longitudinal follow-up visit, and a quarter had long COVID-19. We also identified that pediatric COVID-19 patients had a long-term impact on health-related quality-of-life parameters, specifically in the physical and school domains.^9^

Notably, Brazil has one of the highest mortality rates in pediatric COVID-19. A recent systematic review, using 2020 data, evaluated the magnitude of COVID-19 death and intensive care unit admission in patients aged 0-19 years around the world. Brazil had the highest rate of pediatric death in the world (23 deaths for 1,000,000 children). In comparison, in the United States of America, a country severely impacted by COVID-19, this number was less than 2 deaths/million.^10^ In another survey analyzing children younger than 10 years of age with data up to May 2021, Brazil reported 32 deaths by 1,000,000 children, second only to Peru, with 41 deaths/million.^11^

These relevant and intriguing findings reinforce the need to identify contributing factors associated with poor outcomes in Brazilian children and adolescents with COVID-19. In Brazil, the pandemic began on February 26^th^, 2020, when the first case was reported in São Paulo. The number of cases increased progressively, reaching a plateau from June to September, followed by a decrease by October 2020. The emergence of the SARS-CoV-2 Gamma variant led to a new increase in case numbers by the end of 2020 and reached its peak in March and April of 2021. However, a curve that truly represents the reality of COVID-19 among children and adolescents is lacking, and filtering the curve of the total cases by age would not provide a reliable picture. Since the majority of children and adolescents are either asymptomatic or present mild symptoms, they seldom seek medical attention or testing for SARS-CoV-2 infection. Additionally, in October 2021, Brazil was only the 125^th^ country in a worldwide COVID-19 testing rank.^12^ Therefore, the numbers of pediatric COVID-19 cases in our country are severely underestimated.

To study the pandemic evolution among children and adolescents in Brazil, most studies use the Influenza Epidemiological Surveillance Information System (*Sistema de Informação da Vigilância Epidemiológica da Gripe* – SIVEP-Gripe) database, which includes data from all the patients hospitalized with SARS. The notification of SARS is mandatory in Brazil, and the dataset contains close to 160 features per case, including age, ethnicity, municipality of residency, attending hospital, symptoms, comorbidities, investigation of etiology, treatments, outcomes, and others. The wide range of information provided in this dataset is essential for epidemiologic research on the evolution of the COVID-19 pandemic in Brazil.

Analyzing SIVEP-Gripe data,^13^ our group found 14,638 cases of pediatric COVID-19 SARS cases in 2020, with 1,203 deaths, an 8.2% lethality rate. These deaths represented 0.6% of the total deaths by COVID-19 in Brazil. Studying the deaths, 42% were in children younger than 2 years and 43% in adolescents (10–19 years), with children from 2–10 years being relatively protected. At least one comorbidity was observed in 58% of the deceased patients. Of note, 69% of the deaths occurred in black or mixed-race (brown) patients, 25.5% in whites, 5% in indigenous, and approximately 60% in the North and Northeast regions. It is important to notice that these proportions are not populational. Indigenous people account for barely 0.5% of the Brazilian population, and only 36% of Brazilians live in the North and Northeast regions, for instance.

As previously mentioned, the year 2021 was marked by the spike in COVID-19 cases and deaths from March to May, driven mainly by the spread of the Gamma variant, which lead to the collapse of the health system and the reinstatement of partial containment measures. From January to early September, 17,000 cases of pediatric COVID-19 SARS were reported, with 1,180 deaths, a lethality rate of 6.9%.^14^ Similarly to 2020, in 2021 most deaths occurred either in children younger than 2 years (37%) or in adolescents (50%); among brown people (59%), and in children with at least one previous condition or comorbidity (58%). Interestingly, despite the Gamma variant being more transmissible, there is currently no evidence showing higher morbimortality in children associated with this variant. The increase in the number of deaths was secondary to the increase in the number of cases; proportionally, the death rate in 2021 was lower than in 2020.

The recognition that some groups might be at higher risk for poor outcomes leads to the study of risk factors for pediatric COVID-19 mortality in Brazil. The first noticeable risk factor is age. Indeed, children younger than 2 years and adolescents are at higher risk compared to children from 2–10 years, with mortality following a U-shaped curve that has already been described by other studies.^10^ The higher risk in newborns and infants might be explained by immaturities. In fact, they have immature immune and respiratory systems and are more prone to worse respiratory outcomes, similar to reports for other respiratory viruses, like SRV. In contrast, the impact of preexisting chronic conditions is more relevant for adolescents, since they have had more years to develop and suffer the deleterious effects of chronic diseases.

Comorbidities are an extremely important risk factor for COVID-19 mortality in Brazilian children and adolescents.^15^ In our study,^16^ using SIVEP-Gripe data from 2020, children with more than one comorbidity (asthma excluded) had almost ten times the odds of mortality compared to children with no previous conditions. Individually, most previous conditions were risk factors, with cardiovascular diseases and renal diseases conferring the higher odds of death. Interestingly, asthma was a protective factor, reducing the odds of death by 60%. The role of asthma as a risk or protective factor is still an open debate, but most studies point that the disease is not a risk factor for COVID-19 severity.^17^

Another important risk factor for severity is ethnic vulnerabilities. Oliveira et al.,^18^ using SIVEP-Gripe data from 2020, showed that indigenous children and adolescents were 3.3 times more likely to die than Caucasians. Our study also identified vulnerability among brown patients, with twice the chance of death compared to Caucasians.^16^ These vulnerabilities might be explained due to lower public health system access, worse health indicators, and higher viral exposure. This reality is especially dramatic for indigenous people, leading to shortcomings in socioeconomic and health indexes.^19^ During the 2009 H1N1 influenza pandemic, for instance, the death rate among indigenous was 4.5 times higher than in the general Brazilian population.^20^

Regionality and socioeconomic development are also very important risk factors for pediatric COVID-19 mortality. Children living in the North and Northeast regions have 3.4 times greater chance of mortality compared to those in the South, Center-East, and Southeast regions. Children living in more developed municipalities have 75% less chance of dying compared to those living in less developed cities.^16^ Among other factors, these findings might be related to health care access, as illustrated by the proportion of children who died outside an intensive care unit (ICU) in 2020. In the North and Northeast regions, 36% of COVID-19 pediatric deaths occurred outside an ICU, while in the South, Center-East, and Southeast regions, this proportion was only 25%.^13,14^

The phenomenon of COVID-19 pediatric mortality in Brazil is multilayered and cannot be reduced to a single explanation, but one of its main actors is social inequalities. Vulnerable children are born in worse conditions, eat less nutritious food, have less access to healthcare and a worse control of chronic conditions, all of which lead to a higher risk of mortality not only by COVID-19 but by most health conditions. Unfortunately, the economic effects of the pandemic have widened the socioeconomic gap in Brazil, increasing the number of families in severe poverty. There is also a critical impact on education, especially for vulnerable populations, that were deprived of formal schooling during the pandemic. Of course, Brazil is not the only country in the world with socioeconomic discrepancies, but it is one of the few that has a data collection system solid enough to show these contrasts. The interplay between COVID-19, chronic diseases, and socioeconomic vulnerabilities has led researchers to propose a COVID-19 syndemic rather than a pandemic.^21^ The syndemic theory is based on the interaction of two epidemics in a background of social inequalities that cluster those conditions, worsening their health outcomes.^22,23^ Approaching COVID-19 as a syndemic challenges us to adopt a broader perspective, going beyond biomedical solutions to encompass the context that clusters those diseases and promote their interaction.

In addition, to the structurally rooted social disparities, the COVID-19 pandemic in Brazil was also affected by political controversies that contributed to the crisis. Trying to counterbalance this scenario, Brazil started its vaccination program in January 2021, leading to a vaccination coverage above 50% of the population so far, including the immunization of adolescents. The vaccination effort has led to a consistent decrease in COVID-19 cases and deaths. Pediatricians must be aware that the risks of COVID-19 related complications, including possible long-term consequences, far outweigh the potential risks of very rare adverse events related to COVID-19 immunization, like myocarditis. Therefore, as a reliable source of information for the family, advocating for vaccination is an obligation of healthcare professionals.

In summary, after its peak in the first semester of 2021, we are now in a more comfortable position relative to the number of cases and deaths of pediatric COVID-19, a merit of our vaccination efforts. Socioeconomic factors, ethnicity, regionality, and development play a major role in the outcomes of pediatric COVID-19 in Brazil. Younger children, adolescents, and those with previous chronic conditions are at greater risk.

Thus, what have we learned from this crisis? How can it help us better navigate future outbreaks and pandemics? Science-based, coordinated national policies are the basis to address these conditions and control viral transmission.^24^ Personal protective equipment for health care professionals and the general public should be systemically provided and its use should be encouraged. Diagnostic tests are essential to break the chain of transmission and should be readily available. It is also important to improve internet access and telecom infrastructure, allowing wide access to telehealth and tele-education, minimizing impacts on formal education and cognitive development. Considering that socioeconomic vulnerabilities are a major player on COVID-19 pediatric mortality, vulnerable children and adolescents should be protected, prioritizing health measures and social assistance. It is also never enough to stress the importance of vaccination, the most important measure in controlling the pandemic. Finally, it is essential to talk about mental health, both for children and healthcare professionals. In challenging and unstable times, the implementation of measures to safeguard mental health for children, adolescents, and healthcare professionals is of the utmost importance.^25^ As health care professionals, pediatricians are one of the most trusted sources of information for families and this responsibility must not be taken lightly. By providing reliable, science-based information and actively participating in policy making, we can help society better navigate these troubling times.
